# Sampling from single-cell observations to predict tumor cell growth *in-vitro* and *in-vivo*

**DOI:** 10.18632/oncotarget.22693

**Published:** 2017-11-25

**Authors:** Alexander T. Pearson, Patrick Ingram, Shoumei Bai, Patrick O'Hayer, Jaehoon Chung, Euisik Yoon, Trachette Jackson, Ronald J. Buckanovich

**Affiliations:** ^1^ Section of Hematology/Oncology, Department of Medicine, University of Chicago, Chicago, IL, USA; ^2^ Department of Biomedical Engineering, University of Wisconsin, Madison, WI, USA; ^3^ Magee-Womens Research Institute, University of Pittsburgh, Pittsburgh, PA, USA; ^4^ University of Michigan School of Medicine, Ann Arbor, Michigan, MI, USA; ^5^ Institute of Microelectronics, Science and Engineering Research Council of the Agency for Science, Technology and Research, Singapore; ^6^ Department of Electrical Engineering and Computer Science, University of Michigan College of Engineering, Ann Arbor, MI, USA; ^7^ Department of Applied Mathematics, University of Michigan, Michigan, Ann Arbor, MI, USA

**Keywords:** cancer modeling, ovarian cancer, cancer stem cell, EGFL6, microfluidics

## Abstract

Cancer stem-like cells (CSCs) are a topic of increasing importance in cancer research, but are difficult to study due to their rarity and ability to rapidly divide to produce non-self-cells. We developed a simple model to describe transitions between aldehyde dehydrogenase (ALDH) positive CSCs and ALDH(-) bulk ovarian cancer cells. Microfluidics device-isolated single cell experiments demonstrated that ALDH+ cells were more proliferative than ALDH(-) cells. Based on our model we used ALDH+ and ALDH(-) cell division and proliferation properties to develop an empiric sampling algorithm and predict growth rate and CSC proportion for both ovarian cancer cell line and primary ovarian cancer cells, *in-vitro* and *in-vivo*. In both cell line and primary ovarian cancer cells, the algorithm predictions demonstrated a high correlation with observed ovarian cancer cell proliferation and CSC proportion. High correlation was maintained even in the presence of the EGF-like domain multiple 6 (EGFL6), a growth factor which changes ALDH+ cell asymmetric division rates and thereby tumor growth rates. Thus, based on sampling from the heterogeneity of *in-vitro* cell growth and division characteristics of a few hundred single cells, the simple algorithm described here provides rapid and inexpensive means to generate predictions that correlate with *in-vivo* tumor growth.

## INTRODUCTION

Recent laboratory work has identified a limited subset of ovarian cancer cells with stem cell marker expression. These cancer stem-like cells (CSC) have been found to have unique biologic properties, including increased tumor initiation capacity and, in some cases, chemotherapy resistance [1–4]. Our group and others have reported that aldehyde dehydrogenase (ALDH) activity, alone or in combination with other stem cell markers, identifies CSC in ovarian cancer [5–8]. These ALDH+ cells have increased chemotherapy resistance, increased tumor initiation capacity, and the ability to produce both ALDH+ and ALDH(-) cells [9]. Suggesting a role in disease chemotherapy resistance and disease recurrence, ALDH+ cells are enriched in both patient derived xenografts and primary chemo-refractory tumor specimens [10, 11]. Given these unique properties, CSCs are an important focus in translational research. Understanding how the small CSCs fraction drives self-renewal and tumor growth will provide insights into tumorigenesis.

Despite the potential importance of CSCs, evaluating CSCs has been a challenge. It is difficult to obtain sufficient numbers of primary CSCs for large-scale studies. In addition, primary human CSC engraftment in mice is inefficient and slow, and can take 6-12 months [5]. Similarly, *in-vitro* growth of primary CSCs is hampered by the poor growth in isolation with traditional cell culture media. Growth in “tumor spheres” can be used to enrich CSCs [4], however this assay often requires tens of thousands of cells to replicate analyses and obtaining this number of cells from primary samples can be problematic.

Given the long standing challenges of studying the growth of rare cell populations, mathematical modeling has been used to extrapolate and explain data from experimental studies into a broader understanding of tumor growth dynamics [12–14]. A variety of mathematical modeling approaches have been employed to describe changes in cancer cell states, but each approach has drawbacks. Markov chains have been deployed to model changes in the cell state equilibrium, and are appealing in their ability to generate a unique long term stationary distribution independent of starting state [15–17]. However these models require the problematic assumption that different cell states grow at equivalent rates [18]. A number of separate stochastic processes have been used to model cancer stem cell growth and resistance [19]. Birth/Death processes are one such stochastic method useful for modeling extinction probabilities and steady-state proportions among different cancer states such as CSCs [20, 21]. Multi-state branching processes are a stochastic process that has been deployed to model hierarchical cell-state relationships such as with cancer stem cells [20]. However, theoretical assessment of steady-state behavior can be limited if the observed data do not conform to certain transitional requirements [22–24]; assumptions regarding feedback between states via a mathematical function are often required to account for even small inequalities in transition rates in order to achieve cell-state equilibrium in stochastic models [25–27]. Both ordinary [28–30] and partial [31, 32] differential equation networks have been employed successfully to model changes between different cellular states, and while these modeling networks afford significant flexibility, they often require the estimation of numerous unobservable biological parameters. Finally, cellular automaton and agent-based models offer computational visualization of cellular subtype interactions within a multi-dimensional environment [33–35]. While generally flexible, these models can require advanced computer code and significant computational time to produce results. Furthermore, all of the methods described require the input of a skilled quantitative scientist. The development of a simple, understandable, data-driven method which does not require significant analysis expertise could expand the reach of CSC modeling.

Here we use data gathered from single cell microfluidic culture observations over short time periods to generate an empirical mathematical model that predicts the behavior of full ovarian cancer population over up to 28 days *in-vivo*. We used a single-cell microfluidic culture device to capture, grow, and analyze the division of single cells [36, 37], observing primary ovarian derived CSC in isolation. These devices, via *in situ* live cell stains, also allow for the direct observation of cell divisions and an analysis of the phenotype of progeny cells. As such, self-renewal and asymmetric division potential of live cells exposed to different environmental or treatment conditions can be assessed. Using growth rates and division patterns, we produced CSC and non-CSC simulation-based predictions for larger mixed populations *in-vitro* and *in-vivo*. We show that this simple approach accurately predicts changes in growth associated with the CSC-oriented growth factor EGF-like domain multiple 6 (EGFL6). Our results demonstrate there is a useful relationship between microfluidics events at the single cell level and growth dynamics in larger *in-vitro* and *in-vivo* systems.

## RESULTS

### Monitoring cell growth and division of ALDH+ and ALDH(-) ovarian cancer cells

**Figure 1:**
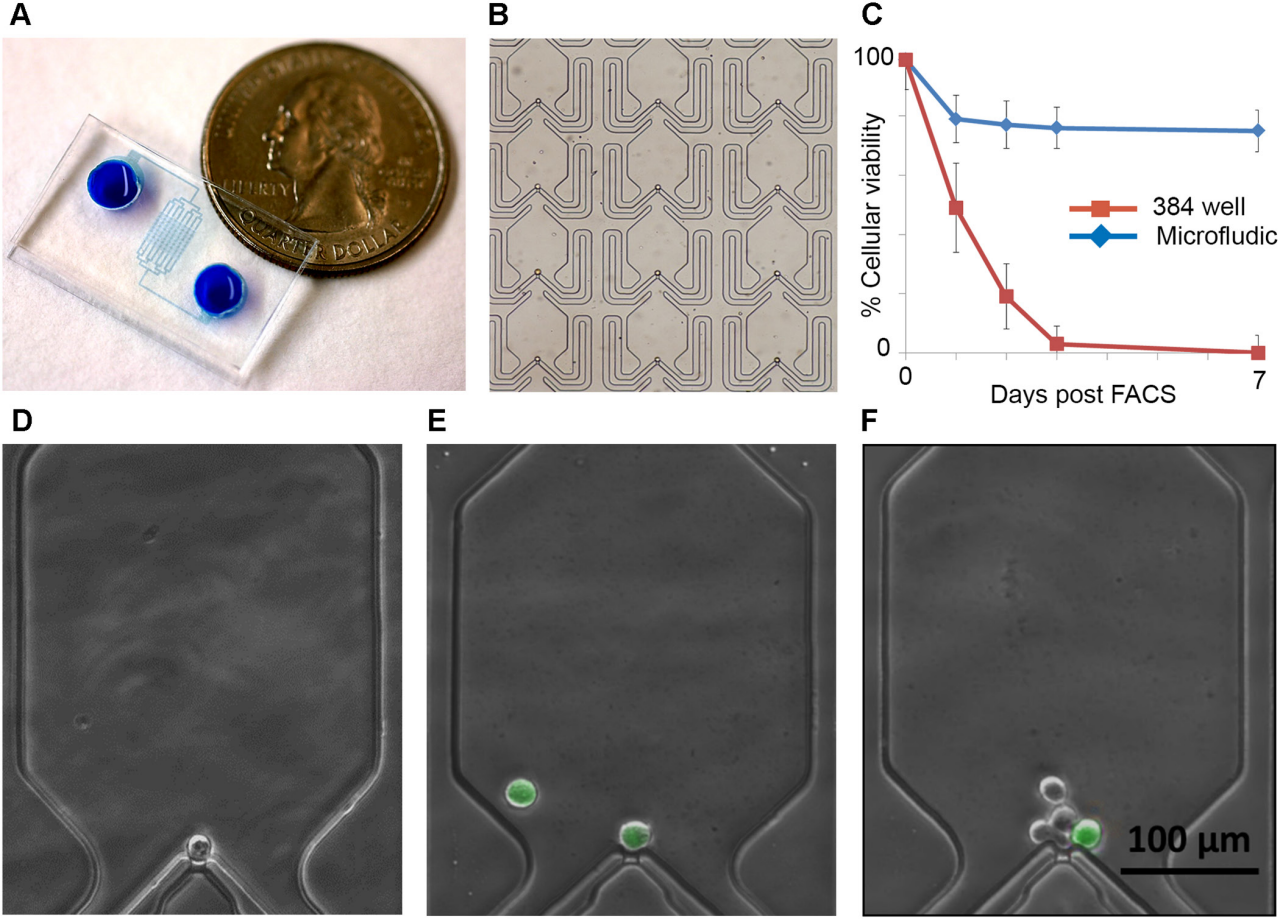
Single cell microfluidics chips allow efficient capture and monitoring of ovarian cancer stem cells **(A)** Photograph of microfluidics chip. **(B)** Magnified image of microfluidics chip array with loaded cells. **(C)** Cellular viability of primary ALDH+ ovarian CSC following FACS in microfluidics culture compared to growth in 384 well plates. D-F. Representative photos demonstrating the ability to track the number and class of progeny from a single captured cell. Green cells are ALDH+; **(D)** represents a live, quiescent ALDH(-) cell, **(E)** indicates an ALDH+ cell that generated a second ALDH+ cell, and **(F)** indicates and ALDH+ cell with multiple ALDH(-) progeny.

While ALDH+ cells represent a small portion of total ovarian cancer cells, they play an important role in chemotherapy resistance and tumor initiation [5, 7]. We used a single cell microfluidic culture method to evaluate the growth of isolated ALDH+ and ALDH(-) cells from the ovarian cancer cell line SKOV3 and a primary ovarian cancer debulking specimens (Figure 1A, 1B). Using passive hydrodynamic structures, an array of microchambers efficiently captures single cells (Figure 1B). While SKOV3 cells demonstrated excellent viability in both traditional and microfluidic culture (90 and >95% viability, data not shown), primary cells demonstrated significantly greater viability in microfluidic culture, surviving and proliferating (Figure 1C). Importantly, within the device the purity of initial of loading, total cell numbers per chamber, and ALDH expression (via the ALDEFLUOR assay) can be directly interrogated. This essential feature allows identification of the cellular state (ALDH+/ALDH(-)) in the captured live cells at initial capture and in the progeny following cell division (Figure 1D–1F).

**Figure 2:**
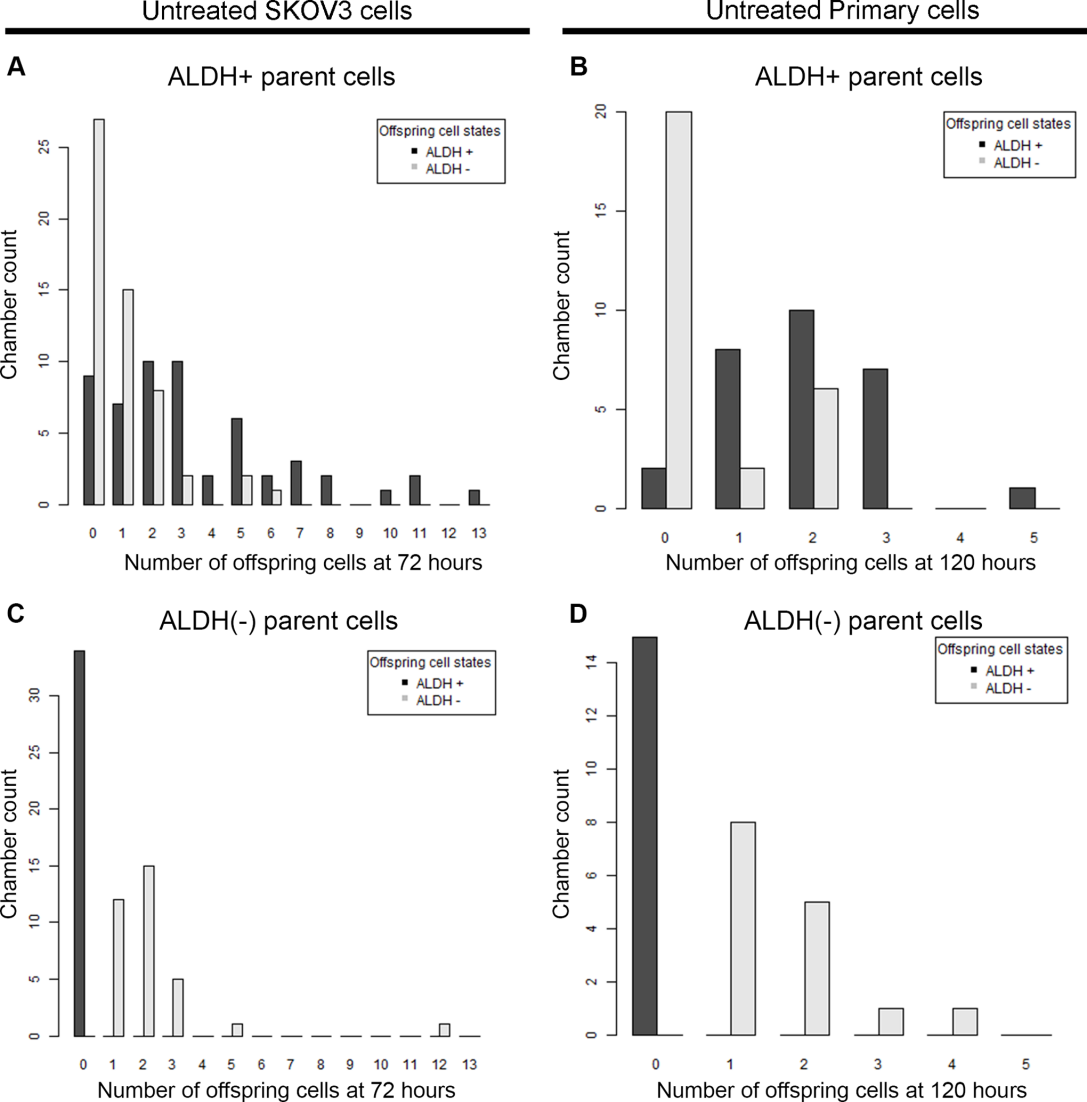
ALDH+ and ALDH(-) cells produce different numbers of offspring in microfluidics chambers ALDH+ and ALDH(-) SKOV3 cells **(A** and **C)** or primary cells **(B** and **D)** were grown in microfluidic culture for 72 or 120 hours respectively. Bar graphs indicate the counts for number of microfluidic chambers (Y axis) with the respective number of progeny cells (X axis) that are either ALDH+ (black bars) and ALDH(-) (grey bars). Live, viable cells which produced no offspring resulted in a zero value on the x-axis. Results are representative of at least two analyses per sample.

After confirming cell growth in the microfluidic device, we evaluated the growth rate of both ALDH+ and ALDH(-) cells. For both SKOV3 and primary cells, ALDH+ cells were more proliferative than ALDH(-) cells; compared to ALDH(-) cells ALDH+ cells were both (i) more likely to divide and, (ii) more likely to generate numerous progeny (Figure 2). ∼12% of SKOV3 ALDH+ cells were quiescent (live but non-dividing) while 35% of SKOV3 ALDH(-) cells were quiescent (p = 0.024). Similarly, for primary cells, 14% of ALDH+ cells were quiescent while 53% of ALDH(-) cells were quiescent (p = 0.018). For SKOV3 cells the average number of cells after 72 hours per dividing single ALDH+ cell was 4.4 whereas the average number of cells after 72 hours per dividing single ALDH(-) cell was 2.2 (p < 0.001). Similarly, for primary cells the average number of cells after 120 hours per dividing single ALDH+ cell was 2.4 whereas the average number of cells after 120 hours per dividing single ALDH(-) cell was 1.7 (p = 0.008).

We also evaluated the ALDH expression of the progeny of cells captured in each chamber. For both SKOV3 and primary cancer cells, ALDH positive cells were observed to generate both ALDH+ and ALDH(-) cells (Figure 1E-1F-1F, 2A, C). In contrast, ALDH(-) cells were observed to only produce ALDH(-) cells (Figure 2B, 2D).

### Developing a cancer cell population growth model and empirical sampling algorithm using *in-vitro* microfluidics device observations

**Figure 3:**
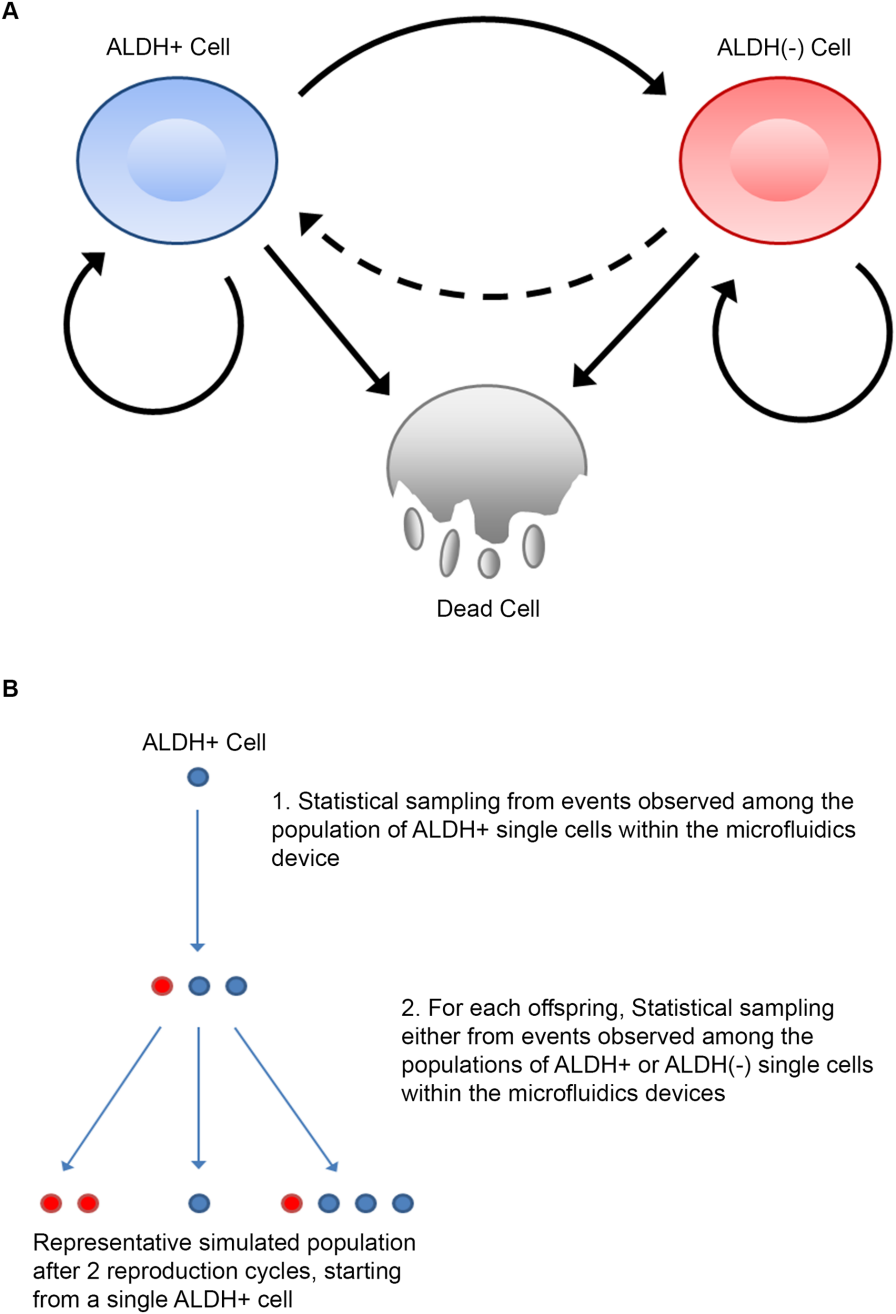
Offspring distribution information defines a map of possible offspring states and empirical predictions of population stem cell state distribution **(A)** Map of possible offspring outcomes based on parent ALDH status. Solid arrows represent transition map between states based on microfluidics data. Dashed arrows represent possible but unobserved transition between ALDH(-) and ALDH+ state. **(B)** Schematic example of one possible set of division outcomes using the sampling algorithm from the equation guide. A hypothetical outcome from an experimental run starting with a single ALDH+ cell over two penalized division cycles is shown.

We conceptualized a simple model of cell state transitions (Figure 3A). In our model cells may undergo one of three fates: symmetrical cell division (producing an offspring of the same type), asymmetric cell division (producing an offspring of the opposite cell type), or cell death. Here, parent cells die in the next time frame with probability g_λ_(t) or survive to divide with probability 1- g_λ_(t). Cell division probabilities are determined using an empirical sampling algorithm that is designed to estimate cell state transitions based on data obtained from *in vitro* microfluidic observations.

In order to determine if we could predict bulk cancer growth with our model using experimental observations of the growth properties of single cells, we iterate the model for cell state transitions in time using the sampling algorithm to select the appropriate transition probabilities at each time step. This sampling algorithm is based on observed cell growth rates in single cell culture and requires a minimum of assumptions to generate its predictions. Briefly, for cells of a specified ALDH status, their number and state of offspring will be drawn from the full spectrum of observed outcomes of ALDH+ and ALDH(-) cells reflected in Figure 2. The non-zero offspring probability distributions define the possible transitions between states (Figure 3A). They also provide a basis for estimating the size and proportion of CSCs in larger populations, by iteratively drawing potential realizations of self-renewal and asymmetric division on a cell-wise basis over many replicates. A schematic of one hypothetical run of the algorithm starting from a single ALDH+ cell is given in Figure 3B. Notably, though no ALDH(-) to ALDH+ transitions were observed, our model would automatically incorporate this transition should future experiments witness de-differentiation events.

### Empirical sampling algorithm

We are interested in the temporal evolution of a population of l distinct cell subtypes, $c_{\lambda }\left(t\right)$, where λ∈(1,⋯,l). These cell subtypes should be observable and quantifiable as they change in time. Each cell is classified into one and only one of the l subtypes. For example, cells could be divided into different categories based on stem cell status. In this manuscript, the l categories would be cells of ALDH+ or ALDH(-) status. The time *t* is observed at multiples of the microfluidics observation period h , so t∈(0,1h,2h,…).

In order to calculate the number of cells of type λ at the next time step t+1, first we define a frequency-histogram-sampled number of additional offspring of cell type *j* produced by cell type *i* at the current time t as by $u_{ij}^{*}$ We can then denote the observed offspring of all l distinct cell subtypes from a single cell of type λ with the vector $u_{\lambda }^{*}=\left[u_{\lambda 1}^{*},u_{\lambda 2}^{*},\ldots ,u_{\lambda l}^{*}\right]$.

Next, we define the probability of parent cell death in the next time interval t+1 as $g_{\lambda }\left(t+1\right)$, determined experimentally for each cell type λ . Then, we define an l length vector of zeros as the representing parent cell death as $O_{\lambda }=\left[0,\ldots ,0\right]$. We can then assign a single sampled realization of our vector of offspring estimates from cell type λ as $q_{\lambda }^{*}=u_{\lambda }^{*}$. Here $q_{\lambda }^{*}$ is a l length sampled vector.

where,

$$q_{\lambda }^{*}=\{\begin{array}{cc} O_{\lambda } & p=g_{\lambda }\left(t+1\right)\\ u_{\lambda }^{*} & p=1-g_{\lambda }\left(t+1\right) \end{array}.$$

Here $q_{\lambda }^{*}$ is a l length sampled vector.

Next, we compute the λ –length row-summed vector of realized sampled outcomes over cells of type λ at time *t*+1 as $r_{\lambda }\left(t+1\right)=\sum_{n=1}^{c_{\lambda }\left(t\right)}$ $q_{\lambda }^{*}$. Here, $r_{\lambda }$ is a 1×l row-vector whose elements are the number of offspring,

$$V\left(t+1\right)=\left[\begin{array}{c} r_{1}\left(t+1\right)\\ \vdots \\ r_{l}\left(t+1\right) \end{array}\right].$$

produced by all the cells of type λ at time *t*.

Therefore, the full l×l realization matrix of cellular growth and state transitions can be written as:

And to calculate the number of cells of type λ at time *t*+1: $c_{\lambda }\left(t+1\right)=\sum_{m=1}^{l}V_{m\lambda }\left(t+1\right)$ .

### The empirical sampling model predicts changes in cell growth in cell lines and primary patient samples

**Figure 4:**
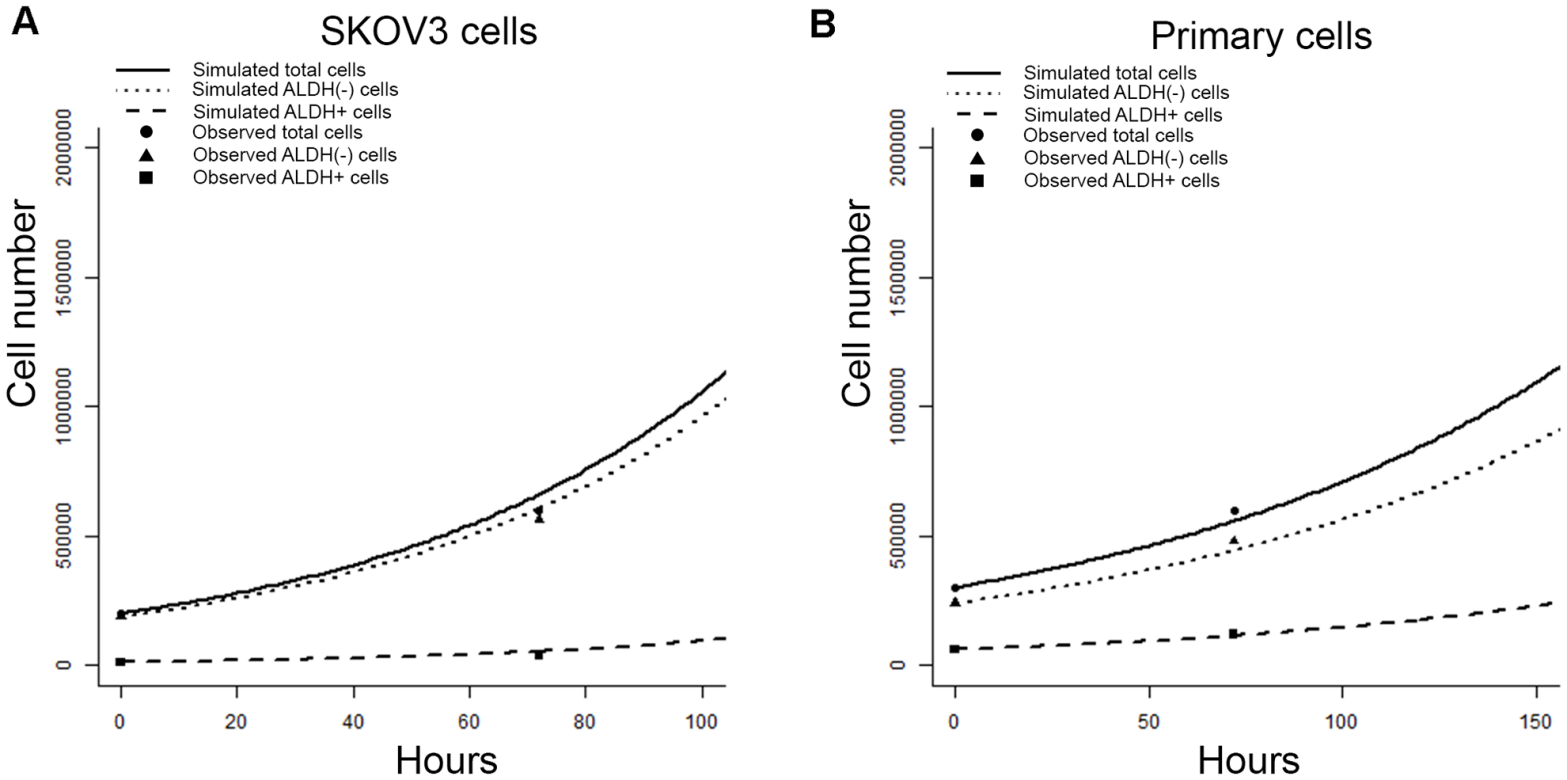
Empirical sampling from microfluidics chamber event observations predicts outcomes in-vitro **(A)** Graph of observed vs. sampling-algorithm predicted ALDH+, ALDH(-), and total cell numbers are generated from 200,000 SKOV3 cells over 72 hours of *in-vitro* growth. **(B)** Graph of model predicted vs. observed, validation ALDH+, ALDH(-), and total cell numbers generated from 300,000 primary cells with validation over 72 hours of *in-vitro* growth. The observed slope changes are an expected reflection of underlying exponential cell population increase.

We next used our empirical sampling algorithm to predict the growth of 200,000 bulk SKOV3 cells (assuming 188,000 ALDH(-) and 12,000 ALDH+ cells at time 0 based on baseline FACS analysis indicating 6% ALDH+ cells). We based the sampling algorithm on the observations from microfluidic culture (Figure 2A) and compared the predicted outcomes of the sampling algorithm to the growth of 200,000 bulk SKOV3 cells grown in traditional cell culture for 72 hours. After 72 hours we counted total live cell number and determined the ALDH+ proportion by FACS. We observed good agreement between observed and predicted cell numbers and ALDH proportion at 72 hours for SKOV3 cells (Figure 4A).

We next assessed the ability of the sampling model to predict the growth of primary cells. We plated 300,000 primary ovarian cells (20% ALDH+ based on FACS), and counted total cell number and ALDH+ percentage after 72 hours. In parallel we used our sampling algorithm assuming 240,000 ALDH(-) and 60,000 ALDH+ cells, as was set up in the *in-vitro* culture for comparison. Once again, we again observed good agreement between observed and model predicted primary cell numbers andALDH proportion at 72 hours (Figure 4B).

### The empirical sampling model predicts changes in cell growth related to CSC targeting growth factors

**Figure 5:**
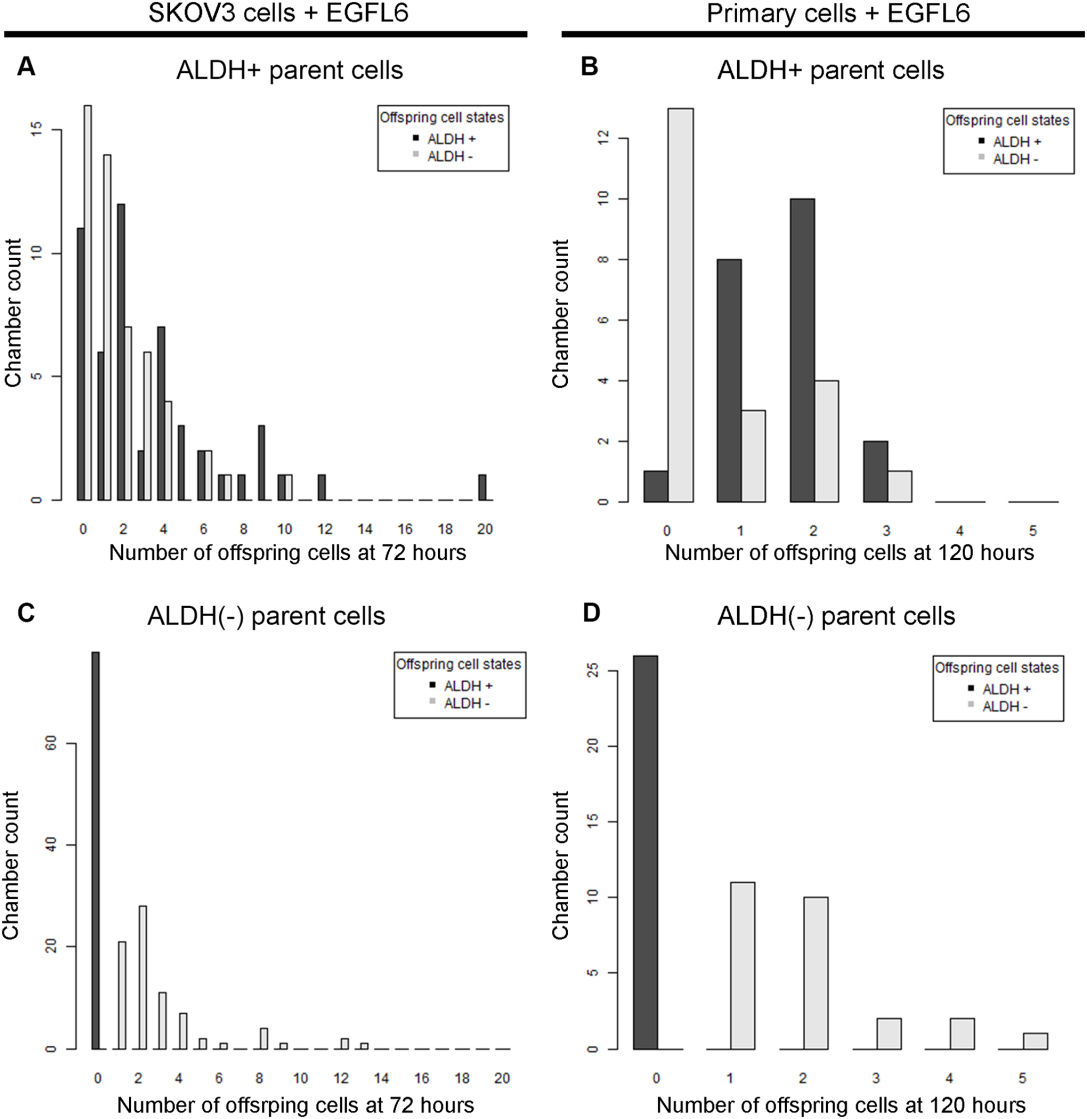
Changes in growth and offspring quantities between ALDH+ and ALDH(-) cells can be quantified in microfluidics chambers response to a CSC targeted growth factor **(A** and **C)** Bar graphs of the number of microfluidic chambers observed (Y axis) with the indicated number of progeny (X axis) from ALDH+ (A) and ALDH(-)(C) parent cells. The quantity of ALDH+ (black) and ALDH(-) (grey) SKOV3 cells grown in the presence of EGFL6are each indicated. **(B** and **D)** Bar graphs of the number of microfluidic chambers (Y axis) with the indicated number of progeny (X axis) from ALDH+ (A) and ALDH(-) (C) primary cells grown in the presence of EGFL6. Cell counts represent the frequency with which a given number of offspring of a given state are observed after 72 or 120 hours for SKOV3 and primary cells, respectively. No ALDH(-) parents were observed to produce ALDH+ offspring. SKOV3 results are representative of triplicate analyses. Primary samples are pooled results from 2 patients in duplicate.

Factors which induce small changes in CSC growth characteristics can significantly alter the growth of bulk cell populations and tumors [38–41]. We next assessed if our microfluidics chip behavior-based modeling schema can predict population growth changes in response to treatment with growth factors. We evaluated the ability of the model to predict the growth changes observed with the exposure of cells to EGFL6. EGFL6 is tumor growth factor produced primarily by tumor endothelial cells [42, 43]. EGFL6 is of particular interest as it acts primarily on ALDH+ cells [39]. We repeated the microfluidic growth assay with ALHD+ and ALDH(-) SKOV3 cells or primary ovarian cancer cells in the presence of absence of EGFL6. After 72 hours, the number and type of daughter cells (ALDH(-) or ALDH+) were scored as described above (Figure 5). EGFL6 treatment was associated with an expansion of ALDH(-) cell self-renewal, with more ALDH(-) cells produced by ALDH(-) parents in both cell line and control cells.

**Figure 6:**
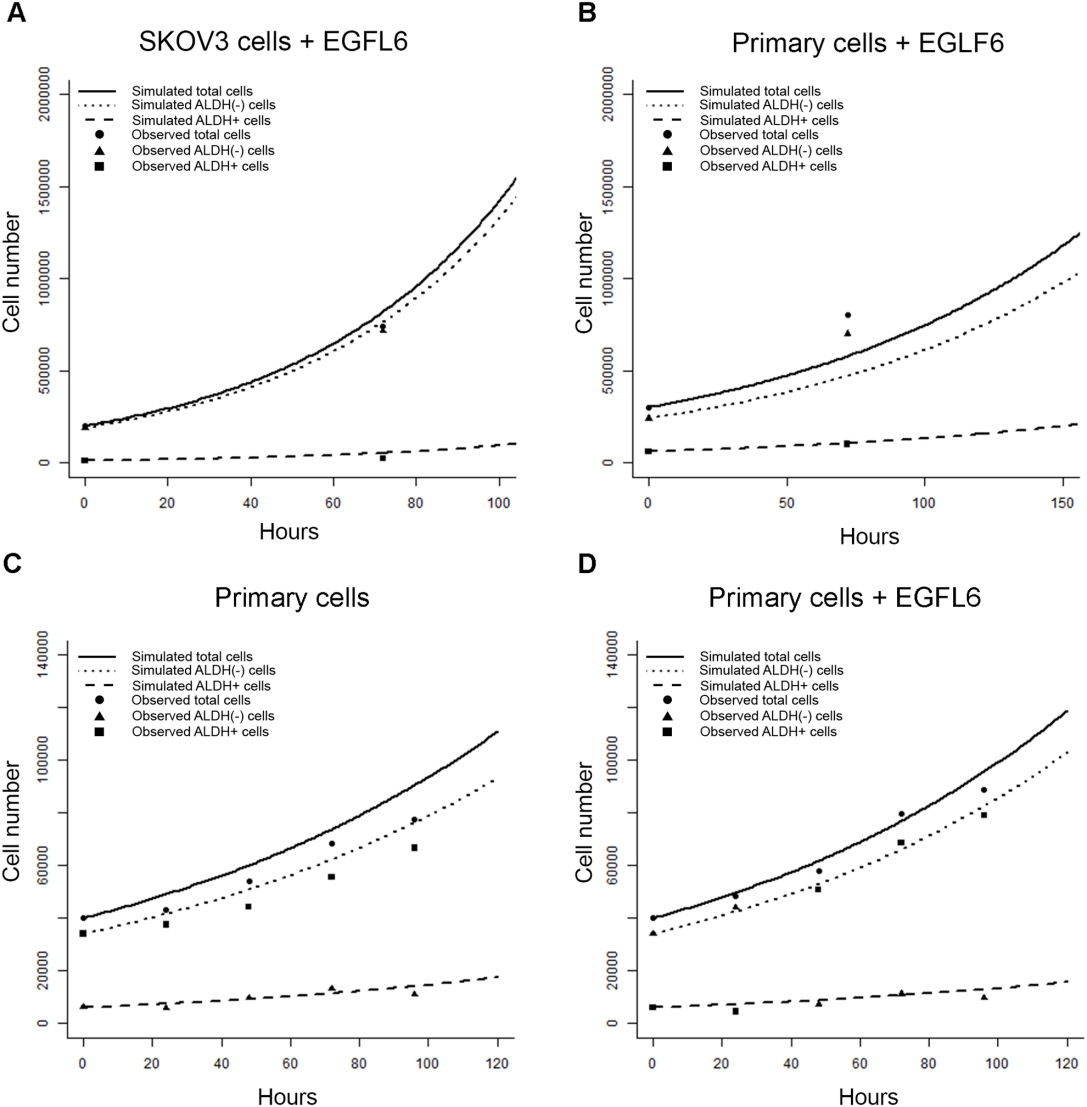
Empirical sampling from microfluidics chamber event observations predicts outcomes after treatment with EGFL6 *in-vitro* **(A** and **B)** Graph of observed vs. model ALDH+, ALDH(-), and total cell numbers generated from SKOV3 (A) or primary cells (B) treated with EGFL6. **(C** and **D)** Graph of repeated primary cell validation measurements on cell quantity and state every 24 hours without (C) and with (D) EGFL6.

In parallel, we evaluated the growth of bulk SKOV3 and primary cells grown with EGFL6. To determine if our empirical sampling based algorithm was able to accurately predict the effects caused by treatment with EGFL6, we ran simulation experiments for SKOV3 and primary ovarian cancer cells based on single cell observations and compared the predictions to bulk growth. Once again, we observed good agreement between observed and predicted total cell numbers and ALDH proportion at 72 hours for both SKOV3 cells (Figure 6A) and primary cells (Figure 6B).

We also evaluated the ability of our algorithm to accurately track primary cells grown with EGFL6 over repeated time points. We ran simulation experiments for primary ovarian cancer cells grown in control media or with treatment with EGFL6. Here we gathered validation measurements on ALDH+ and ALDH(-) cell number experiments every 24 hours for 4 days (Figure 6C, 6D). We again saw good prediction of the validation output by our model, particularly in the ALDH+ cell pool.

### The empirical sampling model predicts *in-vivo* growth

The ability to predict *in-vivo* tumor growth using an *in-vitro* assay would be both time and cost-effective. To investigate the potential of microfluidics growth observations coupled with the sampling model to predict tumor growth under different growth conditions, we conducted a parallel *in-vivo* and in-silica experiment. EGFL6 is expressed primarily in the vasculature and is not expressed by SKOV3 cells, so to assess the effect of EGFL6 *in-vivo*, we initiated tumors using SKOV3 cells co-injected with control hemangioma stem cell (HemSC) derived endothelial cells or HemSC derived endothelial cells expressing EGFL6. Tumor size was measured for palpability through 21 days total. Though the microenvironment is distinct from the ovary, we chose an orthotopic *in-vivo* model for cost and ease of serial measurements. We ran our empirical sampling algorithm, drawing our samples for SKOV3 cell behavior from the observed data in Figure 2A-2B for control cells (SKOV3), and from Figure 6A-6B for EGFL6 treated cells (SKOV3 with HemSC cells expressing EGFL6), starting with 200,000 simulated cells. To compare our simulated ovarian cancer cell outcome numbers to xenograft tumor volume data, we assumed 100,000,000 cells per cm^3^ [44].

**Figure 7:**
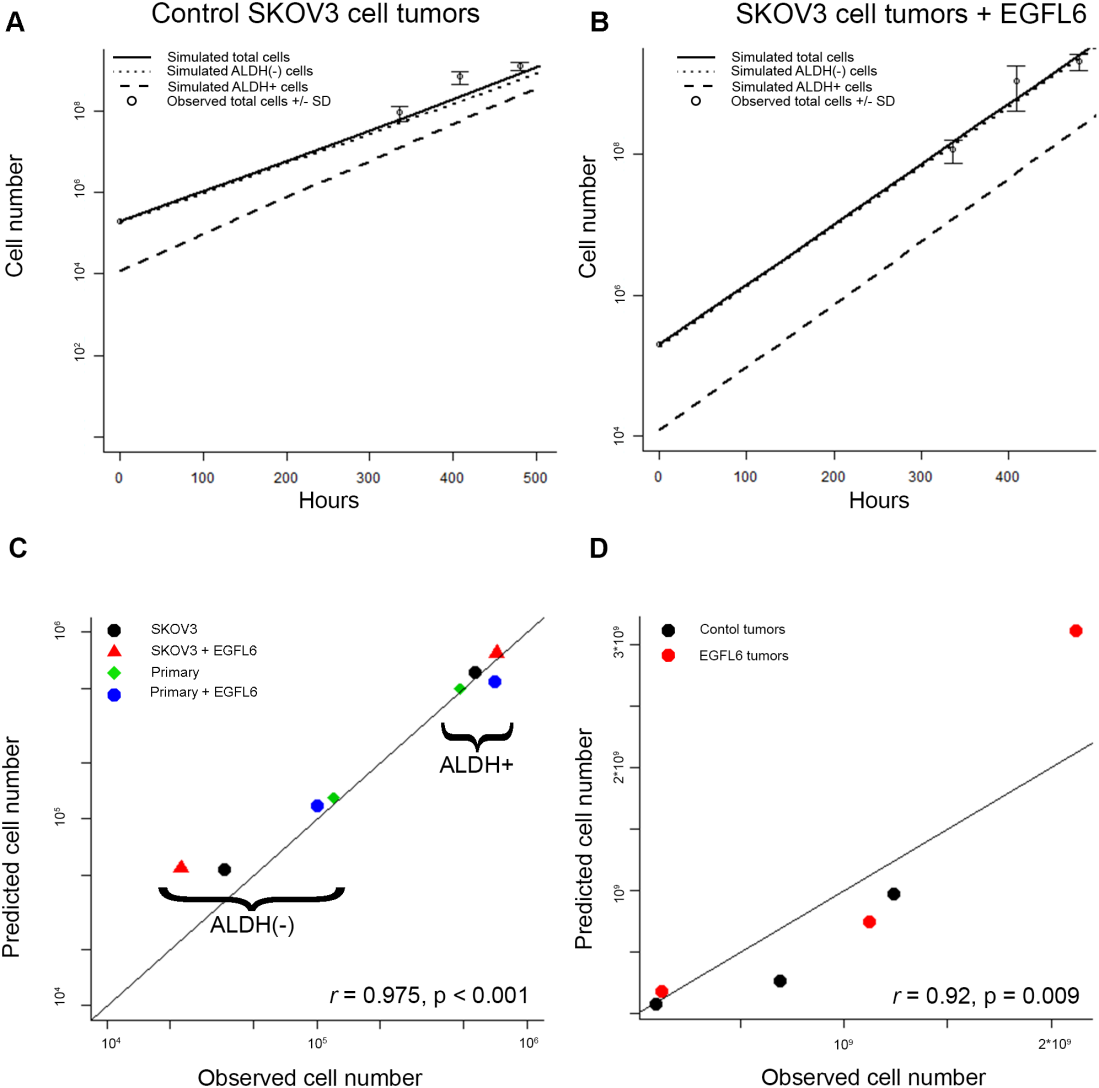
Empirical sampling from microfluidics chamber predicts outcomes *in-vivo* and across multiple experiments **(A** and **B)** Observed and predicted total, ALDH+, and ALDH- cell numbers estimated from *in-vivo* tumors grown in the absence (A) of presence (B) of EGFL6. **(C)** Association between predicted and observed cell number values across all *in-vitro* conditions. **(D)** Association between predicted and observed values across *in-vivo* tumor growth time points.

Our *in-silico* control SKOV3 predictions correlated well with the observed results (Figure 7A). Similarly, the predictions generated from EGFL6 treatment in single cell devices predicted an increased proportion of ALDH(-) cells as well as an increase in total cell numbers (Figure 7B).

Predicted results from our algorithm correlated well with predictions from both *in-vitro* and *in-vivo* experiments. A correlation coefficient calculated for the eight mean observed and eight algorithm-predicted *in-vitro* values showed excellent correlation (*r* = 0.98, p <0.0001, Figure 7C). Similarly, the median *in-silico* predictions correlated very well with the mean observed cell numbers for the xenograft tumor volume data over three observations up to 28 days (r = 0.92, p = 0.009, Figure 7D).

## DISCUSSION

Translational cancer research is a costly and time-consuming endeavor. The implicit requirement for *in-vivo* data during anti-neoplastic drug development mandates expensive mouse (or other mammalian host) experiments. The ability to predict *in-vivo* tumor growth, which takes weeks to months, from small numbers of cells grown *in-vitro* over a period of days could expedite research and significantly reduce costs. To this goal, we have described here a relatively simple single cell system using a few hundred purified CSC and non-CSC and an empirical sampling model that predicts population growth both *in-vitro* and *in-vivo*.

The role of CSCs in tumor biology is an important topic, yet is surrounded in controversy. In particular, controversy exists as to the plasticity non-CSC to attain a CSC state. This is likely to be, at least in part, due to contamination rates (∼1%) associated with standard cellular purification procedures, such as FACS, that are used in many studied [5, 45, 46]. Using the microfluidic single cell culture approach, CSC marker expression can be confirmed in cells after capture and isolation, eliminating the possibility of CSC contamination in non-CSC pools and vice versa. Furthermore, the microenvironment of the microfluidic device (compared to 384 well plates) is more amenable to single cell growth, allowing >95% viability of isolated cell line CSC and >60% growth of primary isolated CSC. Using this device, we observed that ALDH+ cells produce both ALDH+ cells and ALDH(-) cells. In contrast ALDH(-) cells only produced ALDH(-) cells. This supports the possibility of an ovarian CSC hierarchy defined by ALDH expression [5]. Furthermore, the ALDH+ cells produced more offspring on average, suggesting that at least a sub-population of CSCs have a higher reproductive capacity or are capable of rapidly responding to environmental cues to increase cell division. It is important to note that our studies do not rule out “de-differentiation” events, and the presence of these events remains uncertain in light of previous studies [38]. Further studies are necessary to determine if factors such as hypoxia or chemotherapy can promote de-differentiation such that ALDH+ cells are generated from ALDH(-) cells, and to better define quiescent and reproductive subpopulations of CSCs.

This device allows cell growth and CSC marker expression to be assessed in live cells over time in a controlled manner. Additionally, this approach facilitates the identification of outcome information from a multitude of cells simultaneously and under similar conditions. In order to rapidly deploy our system on primary cells, we used the ALDEFLUOR assay instead of an engineered CSC fluorescent gene reporter. With this information, we can begin to construct an understanding of cancer cell type specific events. Using the growth and differentiation information we observed for the ALDH+ and ALDH(-) cell populations in microfluidic culture, we developed an empirical sampling based algorithm to predict the growth of bulk cells *in-vitro* and *in-vivo*.

Our modeling framework is appealing as it is driven by the laboratory data without the extensive mathematical assumptions or parameter estimation for predictive functioning that are inherent in other mathematical modeling techniques. This mechanistic simplicity and transparency can allow for the deployment of our approach by a wide range of researchers. Our algorithm could be applied to markers of interest other than ALDH, including engineered fluorescent reporter genes.

Our empirical sampling framework has produced results supporting a straightforward mechanism to predict changes in cancer growth based on rapid microfluidics experiments. Our results show promising agreement with both *in-vitro* and *in-vivo* results. Importantly, this model functioned both in unmodified populations and in the presence of a growth factor that altered cell states and growth rates. Furthermore, the algorithm corresponded with validation experiments under both cell line and primary cell data where the growth rates and experimental time frames were significantly different. We postulate that predictive accuracy is improved by incorporating stochastic information on differential growth rates and cell transitions between the two cell populations. Despite the successes of the model, further simulations and modeling refinements can improve the approach. In particular, for simplicity in these proof of principle experiments, we used a 2-state model, however this is clearly an over-simplification as there are likely multiple additional cancer cell populations present [5, 38]. Furthermore, important cell-cell interactions are limited in our current microfluidics device. In addition, studies of the ability of this algorithm to predict anti-neoplastic response *in-vivo* would be of great use. We also believe that more realistic prediction functions could be generated by using a continuous time, rather than discrete time, modeling framework. Opportunities for computational modeling refinement will continue as microfluidic technology improves. Single cell co-culture devices may improve our *in-vivo* predictive accuracy by recreating microenvironmental effects as well.

In conclusion, we have generated a simple model to use cell state (CSC/non-CSC) growth to predict the growth of populations of cell *in-vitro* and *in-vivo*. Using proliferative heterogeneity information from small numbers of primary cells, this model can also be used to predict the response of a population of cells to growth factors which alter cell state. This study lays the groundwork for future work potentially combining single cell studies and mathematical models to predict response to therapeutics for translational drug discovery studies and ultimately personalized medicine.

## MATERIALS AND METHODS

### Microfluidics experiments

Microfluidic single cell devices were fabricated using PDMS soft lithography as detailed in [36]; PDMS was thermally aged and soaked in ethanol overnight to remove potentially uncured oligomers. Cells were trypsinized, fluorescence activated cell sorting (FACs) isolated, and loaded into microfluidic devices in supplemented mammary epithelial basal media (MEBM) media as previously described [38] such that ∼80% of chambers contained a single cell based on direct microscopic observation. The remainder of chambers either contained multiple cells or were empty. Direct immunofluorescent (IF) microscopy was used to confirm identity (ALDH+ or ALDH(-)) of the captured cells immediately after capture to prevent FACS contamination. Devices were then incubated at 37°C with 5% CO_2_ for the specified time period. Cells were then restained with ALDEFLUOR (Stem Cell Technologies) within the device after 72 (SKOV3) or 120 (Primary cells) hours based on the differential growth rates of these cell types, and IF microscopy was used to evaluate type of daughter cells (ALDH(-) or ALDH+) produced and total cell numbers. Cell count and ALDH-status scoring were uniformly counted by a single operator. Cellular death rate was estimated by performing flow cytometry sorting on Annexin-V stained and ALDEFLUOR stained cells to give the proportion of apoptotic ALDH+ and ALDH(-) cells.

### Ovarian cancer cells

SKOV3 cells were maintained in RPMI-1640 media, supplemented with 10% FBS with 1% Penicillin/Streptomycin, and cultured in humidified atmosphere of 5% CO2 at 37°C. For primary cells, all tissue was procured after obtaining informed consent, and procurement was approved by the Institutional Review Board of the University of Michigan. Tumors used in this study were stage III/IV high grade serous epithelial ovarian cancer. Tumors were mechanically dissected into single-cell suspensions and isolated on a ficoll gradient as previously described[47]. For ascites, cell pellets were collected by centrifugation; red cells were lysed using ACK buffer (Lonza, Hopkinton, MA, USA), washed, passed through a 40-μm filter, then passed 4 times through a Standard Hub Pipetting needle to isolate single cells [5].

### Murine studies

All animal experiments were conducted in accordance with institutional guidelines of the University of Michigan, and the studies were approved by the University Committee for Use and Care of Animals. SKOV3 cells (chosen as they are a non-EGFL6 expressing cell line) (2x10^5^) were mixed were mixed with EGFL6-expressing human infantile hemangioma stem cells (HemSC,1x10^6^) or equal number of control HemSC. The cells were mixed with Matrigel and injected into the axilla of NSG mice (n=10/group) as previously described [5]. Tumor volumes were monitored overtime and tumor weights obtained at the time of euthanasia.

### EGFL6 production

HEK293 cells were transiently transfected with EGFL6 plasmid using FuGENE 6 reagent (Promega) per protocol in growth medium containing 2% FBS. Supernatant was collected at 36 hours and 72 hours after transfection. Supernatant from empty vector transfected cell was collected as controls. To obtain purified EGFL6, recombinant EGFL6 flag protein was expressed by transient transfection of HEK293 cells and purified with Anti-FLAG M2 Affinity Gel (Sigma). Briefly, cell lysate was loaded onto the FLAG M2 Affinity Gel column under gravity flow on ice, and washed with 10-20 column volumes of TBS. The bound FLAG-EGFL6 fusion protein was eluted with 0.1 M glycine HCl, pH 3.5, into vials containing 20 μL 1 M Tris, pH 8.0 to neutralize pH. Eluted FLAG-EGFL6 fusion protein was used immediately or stored at -80°C in 10% glycine.

### Empirical simulations

Numerical simulations were performed using the statistical program R 3.1.0 [48]. Simulations were performed over 50 iterations, and median values recorded. Starting values for each simulation were chosen to match observed values from experiments.

### Statistical analysis

Comparisons between means were performed using the nonparametric Mann-Whitney U test. Comparisons between proportions were performed using the chi-squared test. Correlation was measured by Pearson’s correlation coefficient *r* [49], and the test version of the statistic [50]. Statistical calculations were performed using the statistical computer program R [48].
